# The Influence of Migration Timing and Local Conditions on Reproductive Timing in Arctic‐Breeding Birds

**DOI:** 10.1002/ece3.70610

**Published:** 2025-01-21

**Authors:** W. B. English, B. Lagassé, S. Brown, M. Boldenow, J. Burger, B. Casler, A. D. Dey, S. Feigin, S. Freeman, H. R. Gates, K. E. Iaquinto, S. Koch, J. F. Lamarre, R. B. Lanctot, C. Latty, V. Loverti, L. McKinnon, D. Newstead, L. Niles, E. Nol, D. Payer, R. Porter, J. Rausch, S. T. Saalfeld, F. Sanders, N. R. Senner, S. Schulte, K. Sowl, B. Winn, L. Wright, M. B. Wunder, P. A. Smith

**Affiliations:** ^1^ Carleton University Ottawa Ontario Canada; ^2^ Department of Biology and Wildlife University of Alaska Fairbanks Fairbanks Alaska USA; ^3^ Manomet Inc Saxtons River Vermont USA; ^4^ Fisheries and Ecological Services U.S. Fish and Wildlife Service Anchorage Alaska USA; ^5^ Division of Life Sciences, Ecology, Evolution, and Natural Resources Rutgers University Piscataway New Jersey USA; ^6^ Fallon Nevada USA; ^7^ Endangered and Nongame Species Program New Jersey Division of Fish and Wildlife Millville New Jersey USA; ^8^ Wildlife Restoration Partnerships Greenwich New Jersey USA; ^9^ U.S. Fish and Wildlife Service, Arctic National Wildlife Refuge Fairbanks Alaska USA; ^10^ National Audubon Society, Audubon Americas Anchorage Alaska USA; ^11^ U.S. Fish and Wildlife Service, Monomoy NWR Hadley Massachusetts USA; ^12^ U.S. Fish and Wildlife Service, Eastern Massachusetts NWR Complex Hadley Massachusetts USA; ^13^ Département de Biologie and Centre d'études Nordiques Université du Québec à Rimouski Rimouski Quebec Canada; ^14^ Canada and Polar Knowledge Canada, Canadian High Arctic Research Station (CHARS) Cambridge Bay Nunavut Canada; ^15^ Migratory Bird Management Division U.S. Fish and Wildlife Service, Alaska Region Anchorage Alaska USA; ^16^ Migratory Bird and Habitat Program U.S. Fish and Wildlife Service Portland Oregon USA; ^17^ Bilingual Biology Program York University Glendon Campus Toronto Ontario Canada; ^18^ Coastal Bend Bays & Estuaries Program (CBBEP) Corpus Christi Texas USA; ^19^ Biology Department Trent University Peterborough Ontario Canada; ^20^ National Park Service Anchorage Alaska USA; ^21^ Ambler Pennsylvania USA; ^22^ Canadian Wildlife Service, Environment and Climate Change Canada Yellowknife Northwest Territories Canada; ^23^ South Carolina Department of Natural Resources McClellanville South Carolina USA; ^24^ Department of Environmental Conservation University of Massachusetts Amherst Amherst Massachusetts USA; ^25^ National Wildlife Refuge System Homer Alaska USA; ^26^ Toronto Ontario Canada; ^27^ Department of Integrative Biology University of Colorado Denver Denver Colorado USA; ^28^ Wildlife Research Division Environment and Climate Change Canada Ottawa Ontario Canada

**Keywords:** Arctic, carry‐over effects, migration, phenology, shorebird, tracking

## Abstract

For birds breeding in the Arctic, nest success is affected by the timing of nest initiation, which is partially determined by local conditions such as snow cover. However, conditions during the non‐breeding season can carry over to affect the timing of breeding. We used tracking and breeding data from 248 individuals of 8 species and subspecies of Arctic‐breeding shorebirds to estimate how the timing of nest initiation is related to local conditions like snowmelt phenology versus prior conditions, measured by the timing and speed of migration. Using path analysis, our global model showed that local and prior conditions have similar effect sizes (Standardised Path Coefficients ± SE of 0.44 ± 0.07 and 0.43 ± 0.07 for snowmelt and arrival timing, respectively), suggesting that both influence the timing of breeding and therefore potentially reproductive output. However, the importance of each variable varied across species. Individuals that arrived later to the breeding grounds did not leave the wintering grounds later, but instead took longer to migrate, potentially reflecting differences in flight speed or time spent at stopover sites. We hypothesise that this may be due to reduced habitat quality at some stopover sites or an inability to adjust their departure timing or migration speed to match the advancing spring phenology in the North. Individuals that migrated longer distances also arrived and nested later. Our results highlight the benefits and potential conservation implications of using a full annual cycle approach to assess the factors influencing reproductive timing of birds.

## Graphical abstract

We used tracking and breeding data from 248 individuals of 8 species and subspecies of Arctic‐breeding shorebirds to estimate how the timing of nest initiation is related to local conditions like snowmelt phenology versus prior conditions, measured by the timing and speed of migration. Using path analysis, we show that local and prior conditions have similar effect sizes, suggesting that both influence the timing of breeding and therefore potentially reproductive output. Our results highlight the benefits and potential conservation implications of using a full annual cycle approach to assess the factors influencing reproductive timing of birds, including carry‐over effects.

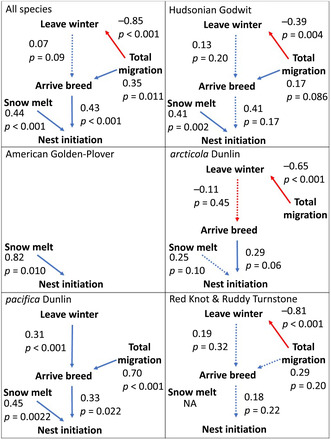

## 1 Introduction

The field of avian ecology is currently in a transition period, facilitated by rapid innovation in tracking technologies (Kays et al. 2015; McKinnon and Love 2018). As we follow avian species throughout the year and around the globe, we can observe how birds move with the seasons, but also how distant regions can be interconnected through their lasting effects on the organisms moving between them (McKinnon and Love 2018; Norris and Marra 2007). With this shift in focus, we have seen repeated examples of the importance of carry‐over effects, where an individual's current abilities are at least partially determined by the conditions and history it has previously experienced (O'Connor et al. 2014).

Marra, Hobson, and Holmes (1998) first highlighted the importance of carry‐over effects using American Redstarts ( *Setophaga ruticilla* ). They found a connection between the quality of an individual's wintering habitat, their subsequent arrival date on the breeding grounds, and ultimately their fitness. Individuals from high‐quality winter habitat produced, on average, more offspring than individuals from low quality habitats (Norris et al. 2004). Thus, understanding the variation in individual reproductive success during the breeding season was impossible in this instance without understanding the conditions experienced by individuals in the non‐breeding season. Subsequent to this paper, carry‐over effects have been found to occur across years (Fayet et al. 2016) across other seasons (Latta et al. 2016), and to affect not only breeding performance but also survival (Duriez et al. 2012). The importance of carry‐over effects may also vary based on other factors like high predation rates (Akresh, King, and Marra 2019), the age or sex of the individual (Drake et al. 2013), or the apparent ability to mitigate effects over following seasons (Conklin and Battley 2012; Ramos et al. 2018).

Measuring the production of fledged young, a metric often used as a proxy for fitness, can be difficult in bird species with precocial offspring such as shorebirds. Chicks are cryptic and may move considerable distances, making determinations of chick survival extremely challenging. However, more easily measured metrics can also serve as reliable indicators of reproductive output. Early nest initiation has been positively linked to higher reproductive performance in many species of Arctic‐breeding shorebirds: early nest initiation has been associated with larger clutch sizes (Kwon et al. 2017; Sandercock, Lank, and Cooke 1999), higher nest survival (McGuire et al. 2020; Weiser et al. 2017), and higher rates of chick growth and survival (Saalfeld et al. 2019). Early breeding may become increasingly important as peak food availability for chicks is projected to advance in response to climate warming (Tulp and Schekkerman 2008).

Local conditions like the timing of snowmelt are known to affect nest initiation phenology in Arctic‐breeding shorebirds (Grabowski et al. 2013; Smith et al. 2010). Shorebirds require snow‐free tundra for nest building, and while small areas of suitable habitat may be available even during periods of high snow coverage, these areas are subject to high predation pressure as they reduce the search area for predators (Meltofte, Høye, and Schmidt 2008; Byrkjedal 1980). Snow cover also affects the availability of feeding areas, and thus the ability of birds to form eggs, as shorebirds are primarily income breeders and derive the nutrients used for egg production from the breeding grounds (Klaassen et al. 2001).

Conditions encountered prior to arrival to the breeding grounds may also influence the timing of nest initiation by affecting when birds arrive (Gill et al. 2001; Smith et al. 2010). Shorebirds are thought to time their departure from the wintering grounds so that they arrive to the breeding grounds at an appropriate time; early‐arriving individuals risk inclement weather and food scarcity, while late‐arrivers must compete with early arrivers for territories and mates, which can prolong the pre‐laying interval (Drent et al. 2003; Morrison, Davidson, and Wilson 2007). However, there is variation in departure timing even among individuals breeding in the same location (Lamarre et al. 2021). Later arrival on the breeding grounds may be the result of later departure from the wintering grounds (Bojarinova et al. 2024; Cooper, Hallworth, and Marra 2017), or may be the result of slower migration (Dossman et al. 2023; Morbey and Hedenström 2020). Variation in the pace of migration can arise because of variation in birds' flight effort, or by the wind conditions experienced during flight (Anderson et al. 2019; Duijns et al. 2019; Senner et al. 2018). However, as refuelling rather than flying occupies the majority of the time spent migrating, refuelling time may contribute more to the observed variation in the duration of migration (e.g., Battley et al. 2012; Johnson et al. 2016). While a large proportion of some species may use a single stopover site, like Red Knots at Delaware Bay, individuals will differ in their use of other stopover locations, and will also differ in where they forage within key sites (Burger et al. 2018; Heller et al. 2022). Differences in refuelling rates can be caused by varying food availability among sites (Acevedo Seaman et al. 2006), or by differences in feeding rate (Murchison, Zharikov, and Nol 2016; Tucker et al. 2019). As individuals get closer to the breeding grounds, they are able to make small adjustments to their arrival timing based on local conditions (e.g., in years with late snowmelt, birds may delay their arrival to their breeding site) (Ely, McCaffery, and Gill 2018); however, these relatively minor adjustments in arrival timing are unlikely to be able to make up for major delays earlier in migration (Aarif et al. 2021; Reneerkens et al. 2020; Swift et al. 2020).

The timing of nest initiation might not be linked to the timing of arrival if later‐arriving birds are able to shorten the interval between arrival and breeding. Population‐level variance in the period between arrival and breeding has been documented across years (Klaassen et al. 2006; Meltofte et al. 2007) and latitudes (Schamel and Tracy 1987), suggesting the potential for some flexibility within individuals; however, a minimum time is likely required assuming that the majority of egg production occurs only after arrival to the general area of the breeding grounds (Roudybush et al. 1979).

Carry‐over effects can appear when individuals are not able to adequately cope with substandard conditions, such as low‐quality wintering (Gunnarsson et al. 2005; Masero et al. 2017) or migration habitat (Baker et al. 2004; Studds et al. 2017). Birds with extreme life histories, such as long‐distance migrants, are thought to be less able to cope with major changes to the resources on which they depend, compared to short‐distance or resident species, as they are already near the limit of what is physiologically possible (Conklin et al. 2017; Gill et al. 2005). Major declines in long‐distance migrant species whose stopover sites have been degraded support the idea that extended migration increases vulnerability to habitat change, and potentially leads to carry‐over effects (Baker et al. 2004; Studds et al. 2017).

In this study, we used geolocator tracking data, field‐based observations of birds' breeding and satellite‐derived estimates of snow cover to determine the extent to which timing of breeding for Arctic‐breeding shorebirds is governed by local conditions versus carry‐over effects from migration. Most studies of carry‐over effects focus on a single species; however, this study includes six species of shorebird, with one represented by three subspecies. We include data from birds breeding across the North American Arctic and sub‐Arctic, from western Alaska to eastern Nunavut; the wintering areas range from eastern Asia to temperate North America to South America. By comparing closely related species, we hope to determine whether differences in key life history traits, such as migration distance, influence the relative importance of local conditions versus migration phenology on the timing of breeding.

We expected that carry‐over effects, measured here as the timing of nest initiation relative to other individuals in the population, may be stronger in longer‐distance migrant species, as these individuals undergo more energetically expensive migrations that could be more sensitive to disruptions, and may be more time‐constrained (Buehler and Piersma 2008). Conversely, species with larger body sizes may not show a strong connection between arrival timing and nest initiation date if they are able to store additional resources that can be used to shorten the pre‐breeding period if they arrive late, or allow them to survive harsh conditions and low food availability in years with late snowmelt (Klaassen et al. 2006; Zhao et al. 2018). We also expected to see some regional differences; for example, birds breeding in western Alaska may be less affected by snowmelt timing than birds breeding in Arctic Canada, because in Western Alaska, snow cover is frequently gone before the birds arrive (Lamarre et al. 2021). We present results from path analyses for all species combined to look for general trends, and single species analyses where sample sizes allowed to look at variation within species and subspecies.

## 2 Methods

### 2.1 Study Species

We used six species of shorebirds to test the relative importance of local versus prior conditions on nest initiation timing. All species in this study are biparental, ground‐nesting, Arctic breeders that migrate to overwinter in temperate or tropical areas (S1). Invertebrates form the majority of their diets, although American Golden‐Plovers ( *Pluvialis dominica* ) and Ruddy Turnstones ( *Arenaria interpres* ) are primarily visual foragers whereas the other species more commonly probe in soft substrates. The majority of the species use coastal habitats during the non‐breeding season, although several use inland areas during migration. Many American Golden‐Plovers winter in grasslands, while some Dunlin ( *Calidris alpina* ) winter at inland wetlands and agricultural areas. See S1 for more detailed individual species accounts.

### 2.2 Geolocator Deployment and Analysis

Migratory routes of the six shorebird species were determined using light‐level geolocation devices. See Table S2 for geolocator analysis methods and classification of wintering versus migration locations. Geolocators were generally deployed on breeding birds at sites across Alaska and northern Canada (Table 1, Figure S1). For most species, the sex of the individuals was unknown, as both sexes incubate in all of the species included in this study. However, Red Knots ( *C. canutus* ) and the majority of Ruddy Turnstones were caught using cannon nets at stopover and wintering sites across the Americas. Geolocators were attached to leg bands, with slight differences in methods between sites. The total mass of the geolocator and leg flag was < 4% of the bird's body mass for Semipalmated Sandpipers ( *C. pusilla* ) and < 2% for all other species.

To estimate an individual's arrival time to the breeding grounds, where continual daylight made arrival difficult to estimate, we took the date of departure from a bird's final stopover site below the Arctic circle, and added the time required to fly from that location to the breeding site, assuming the shortest possible route and a flight speed of 16 m/s, which is a moderate speed for migrating shorebirds (Alerstam and Gudmundsson 1999). While birds may not arrive at the exact date estimated, this method still provides a measure of relative timing while taking into consideration the distance between the last stopover site that provides location data and the breeding site. For species where geolocators were not deployed on the breeding grounds, we were unable to determine breeding location. For these tags we assigned a breeding location based on the approximate centre of the breeding range for *rufa* Red Knot and the eastern North American breeding population of Ruddy Turnstone, and used these locations in our estimates of total migration distance. In other species, total migration distance was determined by using geodesic distances from winter to stopover sites to the final breeding site. This measure was then used to assess whether this trait influenced the relative importance of local and prior conditions on nest initiation.

### 2.3 Nest Initiation Dates and Snowmelt

For birds with geolocators deployed in the Arctic, nest initiation dates for the breeding season following the collection of tracking data were derived by finding nests and estimating age following Liebezeit et al. (2007) and Brown, Lanctot and Sandercock (2014). For birds whose geolocators were deployed away from the breeding grounds, or when nest initiation dates were not estimated at the breeding site, light level measurements were used to estimate nest initiation date, following Burger et al. (2012). Blind tests of this method compared to field‐estimated dates for the onset of incubation found a maximum of 2 days difference between methods across all species.

Timing of snowmelt for breeding sites was derived from MODIS Snow Cover Daily L3 Global 500 m Grid data (Hall and Riggs 2021). For additional information, see S3. Because solar geolocation cannot provide precise location estimates in the 24 h daylight of the Arctic, exact breeding locations were unknown for the species captured away from the breeding grounds. We could not, therefore, determine snowmelt dates for the breeding areas of these species.

### 2.4 Statistical Analyses

We used path analyses to determine the relative influence of migration timing and snowmelt on the timing of nest initiation. Our path analyses included variables representing timing of migration (departure from the final wintering area and arrival to the breeding area) to allow us to assess whether differences in the timing of nest initiation were derived from the timing of departure from the wintering grounds, or from differences in the distance and duration of northward migration. We created a set of directed acyclic graphs (DAG) representing our hypothesised relationships between nest initiation, local conditions (snowmelt) and variables affected by prior conditions (migration timing and distance), based on information from previous studies, following Dale et al. (2015). Each variable was scaled within a species prior to analysis to ensure that effect sizes were comparable across variables.

### 2.5 Model Selection

To understand factors that explained variation in the timing of nest initiation, we developed a model set that included the date of 50% snowmelt and the estimated timing of arrival on the breeding grounds. We started with the most complex model, which included the effect of departure from the wintering grounds on the timing of departure from the last stopover site and nest initiation date, the effect of the timing of departure from the last stopover site on nest initiation date, total migration distance, and the effect of snowmelt timing on nest initiation date. We compared this model to less complex combinations of the same variables, which were selected based on previous literature (for full model set, see Figure S2) (Carneiro et al. 2021; McGuire et al. 2020; Saalfeld and Lanctot 2015; Yohannes et al. 2010). To understand factors that explained arrival to the breeding grounds, we used models that included timing of departure from the wintering grounds and total migration distance. To understand factors that explained departure date from the wintering grounds, we included models testing for an effect of total migration distance. We then broke down the DAGS into linked linear models for path analysis, and used the d‐separation approach to determine which factors most influenced initiation date, either directly or indirectly (Shipley 2009). Unlike structural equation modelling, d‐separation can be used when sample size is small, and when variables are intercorrelated or hierarchical (e.g., multiple individuals within each site) (Shipley 2013). We calculated path coefficients and *p*‐values using linear mixed effects models (function *lmer*; package ‘lme4’, Bates et al. 2015). We modelled species simultaneously, including both species and tag deployment site as random effects. Year was not included in the models because it was confounded with site and species in many cases, and we assumed that differences in snowmelt values would account for much of the yearly variation in nest initiation timing. In some cases, (2 *articola* Dunlin, 12 Hudsonian Godwit, 4 Red Knot, 2 Ruddy Turnstone), two or more years of data were available for the same individual. We present the full data set having found no difference in results when multiple records were excluded.

We conducted the above analyses for all species pooled (except for Red Knot and Ruddy Turnstone), and for individual species (and subspecies in Dunlin) separately where sample size was > 15 unique individuals. In the single‐species models, we included site as a random effect. For Red Knot and Ruddy Turnstone, for which snowmelt values were unavailable, we included tag deployment site and species as random effects.

We ranked models using the *C*‐statistic information criterion, which is equivalent to AICc but uses Fisher's *C*‐statistic, and is produced by d‐separation tests of the path models (Dale et al. 2015; Shipley 2013). We considered models to be competitive with the top model if ∆CICc was within 2 units, after excluding models with uninformative variables (Arnold 2010).

## 3 Results

### 3.1 Timing of Snowmelt, Arrival and Departure

Snowmelt generally occurred earlier at sites in southern and western Alaska and Churchill, Manitoba, compared to northern Alaska and Nunavut, Canada (Table 2). Timing of arrival to the breeding grounds varied by 54 days across species and sites (Table S2). The mean date of arrival generally occurred after the mean date of 50% snowmelt at sites with earlier snowmelt (Table S2). Timing of departure from the wintering grounds was highly variable across species, owing to early departures for American Golden‐Plover and Hudsonian Godwit (Table 2); these species also flew the farthest. Ruddy Turnstone and Red Knot left the wintering grounds latest and had the latest mean nest initiation dates (Figure 2, Table 3, Table S1). *Pacifica* Dunlin had the shortest migration duration, while American Golden‐Plover had the longest (Figure 2). The pre‐breeding interval (time between estimated arrival to the breeding site and nest initiation date) was shortest in *hudsonia* Dunlin (8.0 ± 6.9 days) while American Golden‐Plover had the longest (16.3 ± 6.3 days; Table 3, Table S1).

**TABLE 1:** Summary of geolocator deployment sites, years and sample size by species.

Field site	Time of year	Geographic coordinates	Species	Deployment years	Number of tags
San Antonio Este, Argentina	Winter	S 40.8° W 64.9°	Red Knot	2012	1
Beluga, Alaska, USA	Breeding	N 61.2° W 151.0°	Hudsonian Godwit	2010–2012	40
Panaquatira, Brazil	Winter	S 2.5°, W 44.1°	Ruddy Turnstone	2013–2015	14
Bylot Island, Nunavut, Canada	Breeding	N 72.9°, W 79.9°	American Golden‐Plover	2012–2016	20
Cape Cod, Massachusetts, USA	Migration	N 41.6°, W 69.9°	Red Knot	2010, 2013	4
Cape Krusenstern, Alaska, USA	Breeding	N 67.1°, W 163.5°	*pacifica* Dunlin	2011	10
			Semipalmated Sandpiper	2013	3
Canning River, Alaska, USA	Breeding	N 70.1°, W 145.8°	*arcticola* Dunlin	2011, 2017	5
			Semipalmated Sandpiper	2014	8
Churchill, Manitoba, Canada	Breeding	N 58.6°, W 93.8°	American Golden‐Plover	2013–2015	4
			*hudsonia* Dunlin	2014, 2017	14
			Hudsonian Godwit	2009–2011	6
Coats Island, Nunavut, Canada	Breeding	N 62.8°, W 82.4°	American Golden‐Plover	2015	1
			Semipalmated Sandpiper	2016	2
Delaware Bay, New Jersey, USA	Migration	N 39.1°, W 74.9°	Red Knot	2012–2013	3
			Ruddy Turnstone	2013	1
East Bay, Nunavut, Canada	Breeding	N 64.0°, W 156.5°	Ruddy Turnstone	2011–2012	3
Tampa Bay, Florida, USA	Winter	N 27.5°, W 82.7°	Red Knot	2010–2012	4
Igloolik, Nunavut, Canada	Breeding	N 69.3°, W 81.5°	American Golden‐Plover	2014–2015	6
Ikpikpuk, Alaska, USA	Breeding	N 70.5°, W 154.7°	*arcticola* Dunlin	2014	5
Izembek, Alaska, USA	Breeding	N 55.2°, W 162.8°	*pacifica* Dunlin	2011	17
Kanaryarmiut, Alaska, USA	Breeding	N 61.4°, W 165.2°	*pacifica* Dunlin	2017	4
Manokinak, Alaska, USA	Breeding	N 61.1°, W 165.1°	*pacifica* Dunlin	2011	12
Nome, Alaska, USA	Breeding	N 64.6°, W 165.7°	American Golden‐Plover	2010	1
South Coast Islands, South Carolina, USA	Winter	N 32.4°, W 80.4°	Red Knot	2011	1
Padre Island, Texas, USA	Winter	N 27.3°, W 97.3°	Red Knot	2010–2012	16
Utqiaġvik (Barrow), Alaska, USA	Breeding	N 71.3°, W 156.5°	American Golden‐Plover	2013	1
			*arcticola* Dunlin	2011, 2017–2018	34
			Semipalmated Sandpiper	2014	8

**TABLE 2:** Average date of 50% snowmelt across sites.

Site	Average date of 50% snowmelt (Julian date)	SD (days)	Number of years	50% snowmelt – average arrival time
Beluga	116	6.9	3	−5.9
Bylot Island	165	2.6	6	5.5
Cape Krusenstern	138	7.2	3	−3.6
Canning River	157	4.3	4	7.5
Churchill	144	7.7	9	−5.2
Coats Island	170	n/a	1	20.8
Ikpikpuk	153	2.2	3	3.2
Izembeck	116	2.3	2	−13.3
Kanaryarmiut	115	n/a	1	−14.6
Manokinak	138	n/a	1	3.9
Nome	145	7.3	2	8.3
Utqiaġvik	160	6.4	6	9.5

*Note:* Number of years is the number of years of data that were included in the average, based on the number of years of tracking and nesting data from that site. 50% snowmelt – average arrival time is the difference between the timing of snowmelt and the timing of arrival of birds at that site. Negative numbers indicate that the mean 50% snowmelt date occurred before the mean bird arrival date, while positive number indicate that birds arrived before 50% snowmelt.

**TABLE 3:** Mean timing of migration and breeding by species. *N* = number of individuals of the species; depart winter = mean Julian date of departure from the final wintering site; arrive breed = mean Julian date of estimated arrival to the breeding grounds; migration duration = mean number of days between departure from the final wintering site and arrival to the breeding site; 50% snow to arrival = mean number of days difference between arrival to breeding grounds and date of 50% snowmelt, where negative values signify that individuals arrive before 50% snowmelt occurs; nest initiation = mean Julian date of nest initiation date; pre‐breeding = mean number of days between arrival to the breeding site and nest initiation; total migration distance = mean total great circle distance between the final wintering site and the breeding site, via stopover sites where birds remained for > 48 h, in kilometres. Standard deviations are given for each variable.

Species	*N*	Depart winter ± SD	Arrive breed ± SD	Migration duration ± SD	50% snow to arrival ± SD	Nest initiation ± SD	Pre‐breeding ± SD	Total migration distance ± SD
American Golden‐Plover	36	55.0 (15.2)	150.3 (5.3)	95.8 (15.5)	−11.1 (10.2)	166.1 (8.0)	16.3 (6.3)	13,471.0 (864.7)
*arcticola* Dunlin	45	118.4 (29.6)	150.8 (4.2)	32.4 (30.5)	−7.7 (8.9)	165.3 (6.2)	14.7 (6.6)	6631.7 (723.5)
*hudsonia* Dunlin	17	123.6 (23.11)	149.8 (4.3)	26.2 (23.4)	4.6 (4.2)	157.6 (7.9)	8.0 (6.9)	3328.7 (480.6)
Hudsonian Godwit	46	97.0 (11.7)	125.2 (9.5)	28.2 (7.6)	5.2 (8.6)	135.6 (13.2)	10.4 (8.1)	13,702.0 (822.5)
*pacifica* Dunlin	45	117.8 (10.6)	133.2 (7.9)	15.4 (12.0)	5.1 (10.7)	146.7 (8.4)	14.1 (6.9)	4043.4 (930.0)
Red Knot	73	126.0 (25.4)	159.9 (5.6)	32.3 (25.7)	n/a	172.0 (4.1)	12.1 (5.6)	4930.1 (2674.5)
Ruddy Turnstone	28	135.7 (2.3)	159.1 (8.5)	23.4 (4.0)	n/a	173.0 (7.7)	12.3 (5.6)	8645.3 (795.5)
Semipalmated Sandpiper	29	117.7 (13.8)	153.5 (8.5)	35.8 (9.1)	−6.0 (10.1)	161.0 (5.3)	10.9 (5.7)	9233.4 (1992.8)

### 3.2 Multispecies Model Results

**FIGURE 1:**
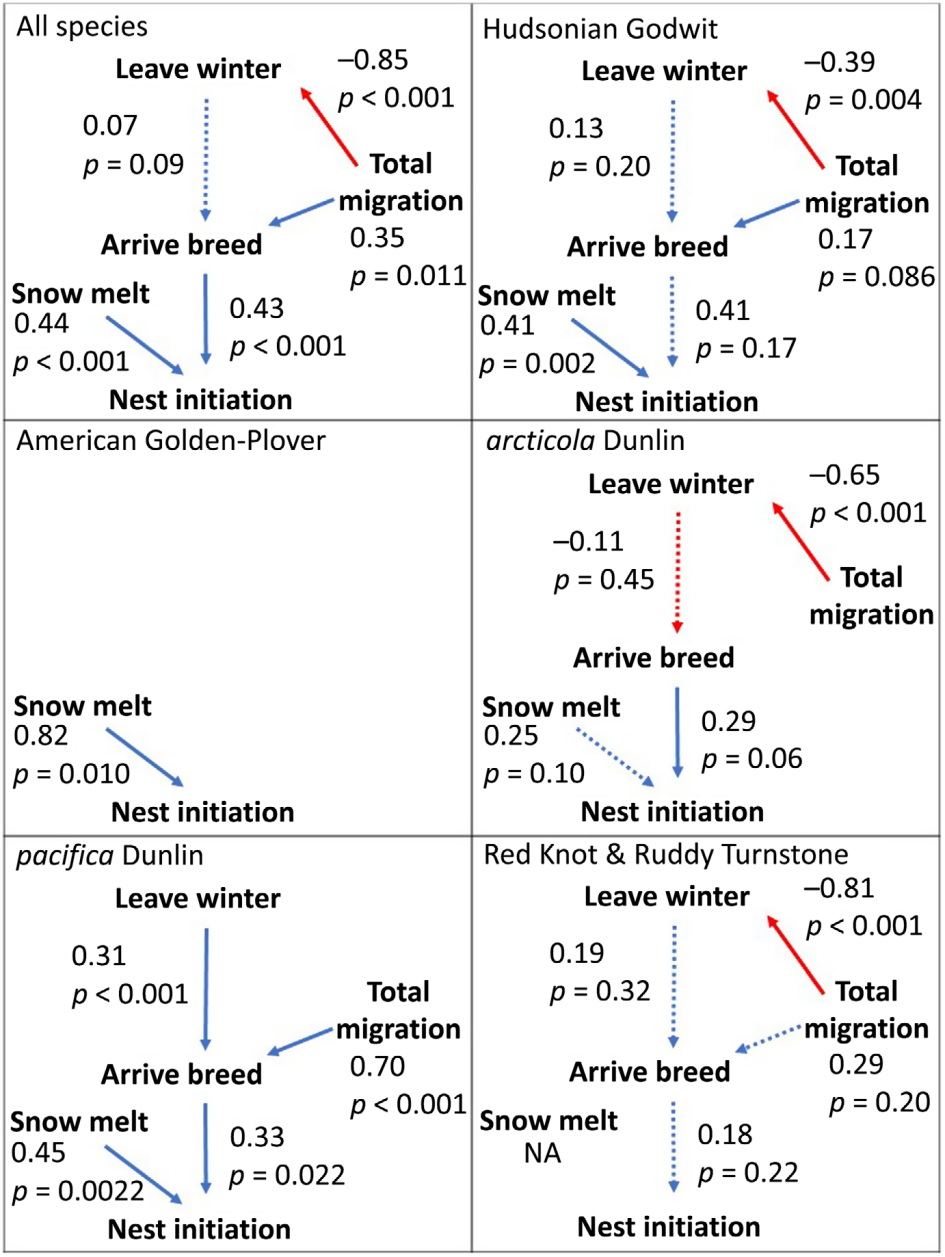
The highest ranked path diagrams for all species combined (except Red Knot and Ruddy Turnstone) and individual species and subspecies. For each linkage in a model, standardised path coefficients and *p*‐values are given. Dashed arrows represent linkages that were present in the top model, but were non‐significant. Solid arrows represent significant linkages. Blue arrows indicate a positive path linkage while red arrows indicate a negative relationship. ‘Leave winter’ is the date an individual left the wintering grounds, ‘total migration’ is the distance in kilometres travelled by an individual between the final wintering site and the breeding site, via identified stopover sites, ‘Arrive breed’ is the estimated date when the bird arrived to the breeding grounds, ‘Snowmelt’ is the date when 50% of ground is snow‐free at the breeding site, and ‘Nest initiation’ is the date when the first egg was laid.

The multispecies models included 202 tracks of 4 species and included species and site as random effects. Standardised Path Coefficients (SPC) and standard errors are given for all model variables in Table S2. The top model had 58% of the support (Table S2), and suggested that snowmelt and the estimated timing of arrival to the breeding grounds both affected the timing of nest initiation to a similar degree (SPC ± SE of 0.44 ± 0.07 and 0.43 ± 0.07, *p*‐values < 0.001; Figure 1). Birds nested later when snowmelt was later, and when the birds themselves arrived later. After controlling for the random effects of site and species, birds that left later from the wintering grounds did not arrive to the breeding grounds significantly later (SPC = 0.07 ± 0.04, *p* = 0.09). Individuals with longer migrations left the wintering grounds earlier (SPC = −0.85 ± 0.17, *p* < 0.001). The top path analysis showed a linkage between migration distance and estimated time of arrival to the breeding grounds (SPC = 0.35 ± 0.13, *p* = 0.011), with birds that travelled longer distances arriving to the breeding grounds later.

### 3.3 Species and Subspecies‐Specific Models

Species and subspecies‐specific model results showed some variation from the multispecies model. Individuals arriving later to the breeding grounds nested significantly later in Semipalmated Sandpiper and all subspecies of Dunlin; for Red Knot, Ruddy Turnstone and Hudsonian Godwit, this pathway was included in the top model but was non‐significant, which can occur if a variable increases model fit but has a *p*‐value > 0.05 due to high variability (Figure 1; Kostenko and Hyndman 2008). This pathway was not included in the top model for American Golden‐Plover. Later snowmelt was related to later nest initiation in all species except *arcticola* Dunlin, where the effect was non‐significant (*p* = 0.10). Later departure from the wintering grounds only resulted in significantly later arrival to the breeding grounds in *pacifica* Dunlin; this pathway was included but non‐significant in the top models for Hudsonian Godwit and *arcticola* Dunlin (Figure 1). Birds travelling farther also arrived at the breeding grounds later, except for American Golden‐Plover and *arcticola* Dunlin. For all species except American Golden‐Plover and *pacifica* Dunlin, a significant negative effect between total migration distance and the date of departure from the wintering grounds was present in the top models (SPC range: −0.39 to −0.85; Figure 1, Table S2).

For Red Knot and Ruddy Turnstone, the top model (CICc weight = 0.52) included the same linkages as the top all‐species model (except for snowmelt, which was unavailable); however, the only significant linkage was the negative relationship between total migration distance and date of departure from the wintering grounds. The next most supported model (CICc weight = 0.48) was identical except that it did not contain the linkage between total migration distance and arrival to the breeding grounds.

## 4 Discussion

Our results demonstrate that the timing of nest initiation in Arctic‐breeding shorebirds is influenced by both local conditions and the timing of arrival to the breeding grounds, with the latter influenced by the duration of individuals' migrations, but generally not their departure dates. Birds with longer migration distances not only left their wintering grounds earlier, but also arrived to the breeding grounds later. Across species, we found generally similar patterns of factors affecting the timing of nest initiation, with some species‐specific differences. Our results demonstrate the importance of both prior and local conditions on the timing of nest initiation, with potential impacts on reproductive success (Kwon et al. 2017; McGuire et al. 2020; Sandercock, Lank, and Cooke 1999; Weiser et al. 2017), in Arctic‐breeding shorebirds.

### 4.1 Local vs. Prior Conditions

The timing of snowmelt is likely to affect the phenology of breeding by influencing the availability of habitat for nesting and the availability of food. Even if sufficient snow‐free areas are available when birds arrive to the Arctic, shorebirds require time after arrival to feed, find mates and establish territories before nests can be initiated. Snowmelt and invertebrate availability are both closely linked to temperature (Brown, Derksen, and Wang 2010; Saalfeld et al. 2019; Tulp and Schekkerman 2008), and in years with late snowmelt, birds are less able to access food, slowing the acquisition of resources needed to produce eggs and preventing early nesting (Meltofte et al. 2006). The time required for resource acquisition and egg development on the breeding grounds may also explain the effect of migration timing on nest initiation. In an array of scolopacid species, Roudybush et al. (1979) found that minimum egg formation time ranged from 5 to 9 days. While the interval between arrival and nesting can be shortened slightly in years with early snowmelt (Klaassen et al. 2001; Meltofte et al. 2007) and can vary with breeding latitude within a species (Schamel and Tracy 1987), the duration between arrival and nesting may already be near to the minimum required for egg production for many individuals (Smith et al. 2010). This suggests that late arrivals have limited ability to make up for delays. Our results highlight the important influence of timing of arrival to the breeding grounds, and not simply local conditions, on the timing of nest initiation.

**FIGURE 2:**
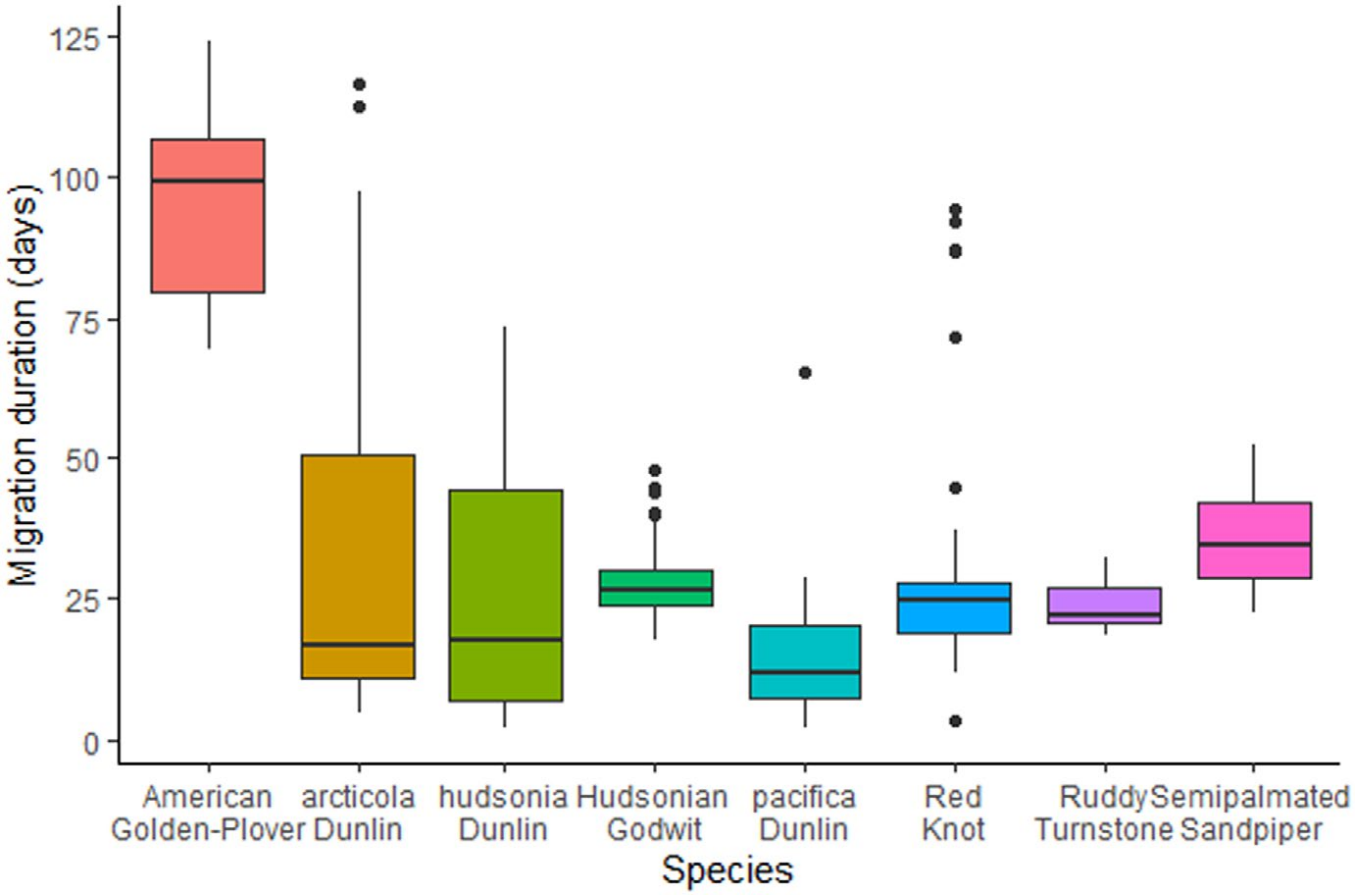
Length and variability of northward migration duration in Arctic‐breeding shorebird species and subspecies. Migration duration is calculated as the time between departure from the final winter site and arrival to the breeding grounds.

Individuals with later estimated arrival times were not late to leave their wintering grounds (accounting for total migration distance), which implies that they migrated more slowly. There was considerable variation in migration duration among individuals, with some species showing more inter‐individual variation than others (Figure 2). Migration speed is governed by factors such as flight speed, route choice and the time needed to refuel between legs of migration. Flight speed is affected both by the flight capability of a bird, and the wind conditions under which it chooses to fly (Anderson et al. 2019; Duijns et al. 2019; Senner et al. 2018). The difference in ground speed between birds experiencing headwinds versus tailwinds has been documented at 11.4 m/s and may exceed this figure in more extreme conditions (Alerstam and Gudmundsson 1999). While individuals that arrived later may have flown more slowly, total migration duration can also be affected by conditions at stopover locations, such as food availability and quality, as these can determine refuelling rates (Rakhimberdiev et al. 2018). The speed at which an individual migrated may therefore also reflect habitat quality along their migration route.

### 4.2 Migration Distance

Individuals with longer migration distances left the wintering grounds earlier, and arrived at the breeding grounds later, on average. While it is unsurprising that birds with longer journeys leave earlier, the reason for later arrival to the breeding grounds is less clear. As long‐distance migrants are thought to be subject to similar selective forces for early nesting, late arrival is unlikely to be beneficial. Long‐distance migrants make departure decisions on their wintering grounds with no knowledge of conditions on the breeding grounds (Gill et al. 2014; Gwinner 1996). Longer‐distance migrants may therefore have less ability to adjust their arrival time to adapt to earlier springs caused by climate change, compared to shorter‐distance migrants (Battley 2006; Both et al. 2010; Doxa et al. 2012; Saalfeld and Lanctot 2017). Long‐distance migrants also tend to make longer non‐stop flights than short‐distance migrants, which are more energetically costly and rely on stopover sites that are consistently rich in food (Anderson et al. 2019; Battley et al. 2012; Warnock 2010). The degradation of such sites is thought to be having negative impacts on long‐distance migrants, with major decreases in nutrition acquisition rates (Baker et al. 2004; Zhang et al. 2019). If long‐distance migrants are timing their wintering ground departures based on a schedule that requires rapid refuelling at high‐quality sites, decreases in habitat quality and food availability at key stopover sites could result in delays in arrival to the breeding grounds that carry over to the timing of nest initiation, and ultimately breeding success (Aharon‐Rotman, Bauer, and Klaassen 2016; Conklin, Lisovski, and Battley 2021; Rakhimberdiev et al. 2018).

### 4.3 Species Differences

In contrast to other species, *pacifica* Dunlin that left the wintering grounds relatively late also arrived at the breeding grounds relatively late. The Pacific flyway used by this subspecies may have less variation in available food resources over time compared to other flyways that are less temperate through the winter (Senner 2012). *Pacifica* Dunlin that leave their wintering sites early may therefore still be able to access enough resources to arrive to the breeding grounds earlier than individuals that leave later. Birds that migrate early on the other flyways would be more likely to encounter snow and cold temperatures that prevent foraging, and a lack of food, preventing further progress (Smith et al. 2020; Thorup et al. 2017). In these flyways, if migratory progress is impeded by poor conditions, birds leaving the wintering grounds later could catch up *en route*.

We found weaker evidence of a link between timing of arrival to the breeding grounds and nest initiation date for Hudsonian Godwit (also shown in Senner et al. 2014), Red Knot and Ruddy Turnstone, in comparison to Dunlin and Semipalmated Sandpiper. The former species differ in having larger body sizes. Although it appears that shorebirds are normally not capital breeders (Klaassen et al. 2001), Morrison and Hobson (2004) used isotopic signatures to show that early‐laid eggs of Red Knot and Ruddy Turnstone contain at least some resources from stopover sites. Species with larger body size could arrive on the breeding grounds with more nutritional reserves, potentially enabling them to shorten the time between arrival and laying by using capital reserves (Tulp et al. 2009). Consistent with this, late‐arriving Black‐tailed Godwits ( *Limosa lapponica* ) nesting in temperate agricultural regions of Europe are known to have shorter pre‐breeding periods than early‐arriving individuals, resulting in a disconnect between the timing of their arrival and nest initiation (Lourenço et al. 2011). Alternatively, species with larger body size may be less affected by the timing of snowmelt if they have the reserves to survive before food is available, which could also cause a disconnect between the timing of arrival and breeding.

For American Golden‐Plover, the linkage between arrival timing and nest initiation was lacking in the single‐species model. In contrast to most of the other species in this study, golden‐plovers are largely visual foragers, and eat more prey from the surface, such as spiders (O. W. Johnson, Connors, and Pyle 2020). Food resources may be available earlier to surface‐feeders compared to species that probe in the ground, as this latter group feeds primarily on species found within the soil, which must be thawed for access (Baker et al. 2020; Hicklin and Gratto‐Trevor 2020; Walker et al. 2020; O. W. Johnson, Connors, and Pyle 2020). Golden‐plovers arrived to their breeding grounds early relative to snowmelt compared to the other species (Table 3), and may be ready to breed as soon as snowmelt conditions allow. However, while golden‐plover were among the earliest arriving species at a site in western Alaska, the opposite was found in the eastern Canadian Arctic (Ely, McCaffery, and Gill 2018; unpublished data).

### 4.4 What Are ‘Wintering Areas’?

American Golden‐Plover left the wintering grounds far earlier than other species, and spent considerably longer on migration. This analysis was affected by the criteria that we used to designate a site as ‘wintering’ or ‘stopover’. In this species, and to a lesser degree in some others, some individuals made major northward movements during the season designated as winter, resulting in a shorter spring migration. Similar large‐scale movements during the non‐breeding season have been recently documented in other groups ranging from thrushes to swallows to phalaropes (Heckscher et al. 2011; Stutchbury et al. 2016; van Bemmelen et al. 2019). The increasing emphasis on full annual cycle studies and carry‐over effects may therefore necessitate further thought about how we differentiate wintering and stopover sites.

### 4.5 Assumptions and Future Directions

This study is based on a tracking dataset that is unprecedented in its number of individuals and species, allowing comparisons across species and covering a large geographic area. However, certain biases may be present that could affect our results. Within a site, geolocators were often all deployed in the same year, making it impossible to disentangle some year and site/species effects. Geolocators are also intrinsically biased given that they cannot distinguish sites within the Arctic during periods of 24‐h light, and only provide data on birds that have returned to be recaptured (Lisovski 2018). As our study centred on nest initiation date, we only included individuals that attempted to breed; therefore, we did not assess the effect of prior or local conditions on breeding propensity, although these are likely present (Burger et al. 2022; McGuire et al. 2020) and might affect the relative importance of each factor. In most cases, we did not know the sex of the individual, therefore we could not account for variation caused by the effect of sex on arrival timing. Protandry is often observed in species where males defend a territory (Gunnarsson et al. 2006; Morbey, Coppack, and Pulido 2012), and has been seen in shorebirds, however, not in all species (Wright et al. 2022). The conclusions we have drawn are based on the best data available, but we acknowledge that there may be effects from the assumptions made and the methods used. Other types of tracking technology that transmit to satellites and provide more accurate data, especially within the Arctic circle (e.g., PTT and GPS tags) have the potential to reduce the need for these assumptions, however the size and location frequency of these tags currently prevents their use on some of the smaller species (Lahoz‐Monfort and Magrath 2021).

### 4.6 Summary

Reduced breeding success is thought to be a contributing factor to the major declines that are occurring in many shorebird species across the globe (Beale, Dodd, and Pearce‐Higgins 2006; Boyd and Piersma 2001; Murray et al. 2018; Ottvall and Härdling 2005). While conditions on the breeding grounds can have a direct effect on reproductive success (Flemming et al. 2019), conditions experienced during the non‐breeding season have also been implicated in variable reproductive success, through carry‐over effects (Baker et al. 2004; Boldenow 2018; Lamarre et al. 2017). Here, we demonstrate the linkage between migration timing, distance and duration, and the timing of breeding, with assumed consequences for reproductive success. Understanding the relative effects of local and prior conditions on Arctic shorebird breeding behaviour and reproductive success is vital for determining the ultimate causes of population declines in shorebird populations, and where and when conservation actions are required to address the threats.

## Author Contributions

**W. B. English:** conceptualization (lead), data curation (lead), formal analysis (lead), funding acquisition (equal), methodology (lead), writing – original draft (lead), writing – review and editing (lead). **B. Lagassé:** data curation (equal), formal analysis (equal), funding acquisition (equal), methodology (equal), software (lead), writing – review and editing (supporting). **S. Brown:** funding acquisition (equal), investigation (supporting), methodology (supporting), project administration (supporting), resources (supporting), writing – review and editing (supporting). **M. Boldenow:** funding acquisition (supporting), investigation (supporting), methodology (supporting), project administration (supporting), writing – review and editing (supporting). **J. Burger:** funding acquisition (supporting), investigation (supporting), methodology (supporting), project administration (supporting), resources (supporting), writing – review and editing (supporting). **B. Casler:** funding acquisition (equal), investigation (supporting), methodology (supporting), project administration (supporting), resources (supporting), writing – review and editing (supporting). **A. D. Dey:** funding acquisition (supporting), investigation (supporting), methodology (supporting), project administration (supporting), resources (supporting), writing – review and editing (supporting). **S. Feigin:** funding acquisition (supporting), investigation (supporting), methodology (supporting), project administration (supporting), resources (supporting), writing – review and editing (supporting). **S. Freeman:** funding acquisition (supporting), investigation (supporting), methodology (supporting), project administration (supporting), resources (supporting), writing – review and editing (supporting). **H. R. Gates:** funding acquisition (supporting), investigation (supporting), methodology (supporting), project administration (supporting), resources (supporting), writing – review and editing (supporting). **K. E. Iaquinto:** funding acquisition (supporting), investigation (supporting), methodology (supporting), project administration (supporting), resources (supporting), writing – review and editing (supporting). **S. Koch:** funding acquisition (supporting), investigation (supporting), methodology (supporting), project administration (supporting), resources (supporting), writing – review and editing (supporting). **J. F. Lamarre:** data curation (supporting), funding acquisition (supporting), investigation (supporting), methodology (supporting), project administration (supporting), resources (supporting), writing – review and editing (supporting). **R. B. Lanctot:** funding acquisition (equal), investigation (supporting), methodology (supporting), project administration (supporting), resources (supporting), writing – review and editing (supporting). **C. Latty:** funding acquisition (supporting), investigation (supporting), methodology (supporting), project administration (supporting), resources (supporting), writing – review and editing (supporting). **V. Loverti:** funding acquisition (supporting), investigation (supporting), methodology (supporting), project administration (supporting), resources (supporting), writing – review and editing (supporting). **L. McKinnon:** funding acquisition (supporting), investigation (supporting), methodology (supporting), project administration (supporting), resources (supporting), writing – review and editing (supporting). **D. Newstead:** funding acquisition (supporting), investigation (supporting), methodology (supporting), project administration (supporting), resources (supporting), writing – review and editing (supporting). **L. Niles:** funding acquisition (supporting), investigation (supporting), methodology (supporting), project administration (supporting), resources (supporting), writing – review and editing (supporting). **E. Nol:** funding acquisition (supporting), investigation (supporting), methodology (supporting), project administration (supporting), resources (supporting), writing – review and editing (supporting). **D. Payer:** funding acquisition (supporting), investigation (supporting), methodology (supporting), project administration (supporting), resources (supporting), writing – review and editing (supporting). **R. Porter:** formal analysis (supporting), investigation (supporting), methodology (supporting), writing – review and editing (supporting). **J. Rausch:** funding acquisition (supporting), investigation (supporting), methodology (supporting), project administration (supporting), resources (supporting), writing – review and editing (supporting). **S. T. Saalfeld:** funding acquisition (supporting), investigation (supporting), methodology (supporting), project administration (supporting), resources (supporting), writing – review and editing (supporting). **F. Sanders:** funding acquisition (supporting), investigation (supporting), methodology (supporting), project administration (supporting), resources (supporting), writing – review and editing (supporting). **N. R. Senner:** data curation (supporting), funding acquisition (supporting), investigation (supporting), methodology (supporting), project administration (supporting), resources (supporting), writing – review and editing (supporting). **S. Schulte:** funding acquisition (supporting), investigation (supporting), methodology (supporting), project administration (supporting), resources (supporting), writing – review and editing (supporting). **K. Sowl:** funding acquisition (supporting), investigation (supporting), methodology (supporting), project administration (supporting), resources (supporting), writing – review and editing (supporting). **B. Winn:** funding acquisition (supporting), investigation (supporting), methodology (supporting), project administration (supporting), resources (supporting), writing – review and editing (supporting). **L. Wright:** formal analysis (supporting), investigation (supporting), methodology (supporting), writing – review and editing (supporting). **M. B. Wunder:** funding acquisition (supporting), investigation (supporting), methodology (supporting), project administration (supporting), resources (supporting), writing – review and editing (supporting). **P. A. Smith:** conceptualization (equal), formal analysis (supporting), funding acquisition (equal), investigation (equal), methodology (equal), project administration (equal), resources (equal), supervision (lead), writing – original draft (supporting), writing – review and editing (equal).

## Ethics Statement

All captures, tagging and nest monitoring were done under appropriate permits from the relevant federal, state, territorial and regional governments, following protocols approved by institutional animal care and use committees.

## Conflicts of Interest

The authors declare no conflicts of interest.

## Supporting information


Data S1

## Data Availability

Data will be available in the Dryad digital repository if manuscript is accepted.
