# Supplementary material for: The Influence of Migration Timing and Local Conditions on Reproductive Timing in Arctic‐Breeding Birds
Source: Ecol Evol. 2025 Jan 21;15(1):e70610. doi: 10.1002/ece3.70610 (PMC11750415; doi:10.1002/ece3.70610)
Supplement: Supplementary file 1 — Data S1 [file ECE3-15-e70610-s001.zip › supplementary material_edited_clean.docx]

Supplementary materials

S1: Species Accounts

American Golden-Plovers (*Pluvialus dominica*) have a broad breeding distribution across the Nearctic, from western Alaska east to Baffin Island, and as far south as Churchill, MA. Most birds overwinter at inland grassland sites in Uruguay, Brazil and Argentina. Southward migration generally consists of long, non-stop flights that are often over-ocean (e.g. eastern USA to northeast South America) followed by a northward flight through the middle of the Americas. Stopover sites vary but are often inland in grasslands or farm fields. American Golden-Plovers are monogamous and both parents incubate and care for chicks (Johnson et al., 2020). American Golden-Plovers are estimated to have declined by more than 70% in the last 50 years (Smith et al., 2023). (Andres et al., 2012).

Dunlin (*Calidris alpina*) are a small to medium shorebird that breeds across both the Nearctic and Palearctic. Multiple subspecies exist, and differ mainly in range, although some show subtle morphological differences. Three subspecies are included in this study: *arcticola* Dunlin that winter in coastal areas of eastern Asia and breed on the North Slope of Alaska, *pacifica* Dunlin that winter mainly on the Pacific coast of North America between California and British Columbia, although some use inland sites in the southern extent of their range, and breed in western Alaska, and *hudsonia* Dunlin that winter in the southeast United States and along the Gulf of Mexico and breed on the coast of Hudson Bay (Warnock & Gill, 2020; Wright et al., 2022). Dunlin are unusual among shorebirds as they start flight feather moult while breeding, and generally finish it at a northern stopover site before completing their migration south. Dunlin are monogamous and both parents share incubation and chick care, although females generally leave the brood earlier (Warnock & Gill, 2020). Arcticola Dunlin have declined in the past several decades, while *pacifica* and *hudsonia* populations appear stable (Andres et al., 2012; Weiser et al., 2020).

Hudsonian Godwits (*Limosa haemastica*) are a large, long-billed shorebird. Five disjunct breeding populations are known, in western Alaska, south central Alaska, northern mainland Northwest Territories and two regions of Hudson Bay. Migration consists of long, non-stop flights between specific stopover areas including central Canada and northeastern South America; most birds winter along the coasts of southern Chile and Argentina. Incubation and chick care is shared equally by both parents (Walker et al., 2020). Hudsonian Godwits appear to have declined by more than 90% in the past fifty years (Smith et al., 2023)

Red Knots (*Calidris canutus*) are medium-sized shorebirds, although they are the largest sandpiper found in the Americas. Three subspecies are found in North America: rufa Red Knot breed in the Arctic Archipelago and show considerable variation in the latitude at which they winter. Many individuals are long-distance migrants and winter as far south as Tierra del Fuego in Argentina, while others winter as far north as New England. Coastal areas are almost exclusively used during winter (Baker et al., 2020). The remaining two North American subspecies are not included in this study. Red Knot are known for their long distance, non-stop flights, and reliance on specific stopover sites such as Delaware Bay. Both parents incubate and care for chicks, although females generally leave only a few days after hatch (Baker et al., 2020). *Rufa* Red Knots have undergone rapid declines of over 90% in the past five decades, and while less data is available, the other North American subspecies (*roselaari*) also appear to be declining (Andres et al., 2012; Smith et al., 2023).

Ruddy Turnstones (*Arenaria interpres*) are a medium-sized shorebird with a pan-Arctic breeding distribution, generally in coastal areas. In the North American Arctic, they breed from western Alaska east to Baffin Island, extending as far north as Ellesmere Island, making them among the northernmost breeding shorebird species. Wintering birds are found exclusively in coastal areas, along both the Pacific and Atlantic coasts and ranging from the continental USA to southern South America (Nettleship, 2020). Two subspecies breed in North America but are hard to distinguish; based on range the birds in this study are likely the *morinella* subspecies, which breed in the central North American Arctic and winter on the Pacific and Atlantic coasts from the United States to South America. Both parents incubate and care for chicks, with females leaving the brood somewhat earlier. Ruddy Turnstones are known for their active and aggressive nest defence, and will dive-bomb predators (Nettleship, 2020). Migration and breeding monitoring suggest that Ruddy Turnstones have undergone substantial declines of more than 70% in the past fifty years (Andres et al., 2012; Smith et al., 2023).

Semipalmated Sandpipers (*Calidris pusilla*) are among the smallest shorebirds, with some individuals under 20g when fat stores are low. This species breeds across the Nearctic, as far north as Baffin Island and Northern Alaska, and as far south as southern Hudson Bay. Breeding populations differ in migration route and wintering area, although there is considerable overlap. Birds breeding in the Eastern Canadian Arctic generally use a more coastal migration route to reach northeastern South America, while birds breeding in the central Arctic and Alaska are more likely to use the central flyway and winter in Central America and western South America. Semipalmated Sandpipers are monogamous and both parents incubate; females generally leave shortly after hatch and males perform the remaining parental care (Hicklin & Gratto-Trevor, 2020). Semipalmated Sandpipers appear to have declined by more than 60% in the last five decades, with steeper declines seen in the eastern breeding population, which winters in northeast South America, and less change in the Alaska-breeding population (Andres et al., 2012; Smith et al., 2023).

S2: Geolocator Processing

We used geolocator data to identify the timing of departure dates from the wintering grounds, and arrival to the breeding grounds. Geolocators were processed following Lagassé et al. (2022) using FLightR (Rakhimberdiev et al., 2017) after preprocessing with GeoLight following Lisovski et al. (2020). Periods where an individual’s position was fixed for >48 hours were considered stationary locations. For processing with GeoLight, we defined the minimum threshold level signifying dusk and dawn by using light levels that remained above ambient night-time light levels, which varied by location.

FLightR requires that a parameter for the maximum distance travelled between twilights be set to limit subsequent potential locations, with a default value of 1500 km. For most birds, this default value was used. For species such as Red Knot that are known to make long non-stop flights (during which time light signals are often disrupted if the birds’ legs are shaded by feathers), the maximum distance allowed to be travelled between two subsequent twilights was increased to a maximum of 6000 km. This was likely necessary because the geolocators were tucked into the bird’s feathers during flight, preventing twilights from being measured during long-distance flights.

We calibrated light-levels of each geolocator using rooftop calibration when available, followed by (in order of preference): in habitat calibration, rooftop calibration from tags of the same model deployed on the same species, and the “find.stationary periods” function (package “FLightR”; Rakhimberdiev et al., 2017). Following the first calibration, we generated a second calibration using the longest stationary period identified in the first run as the new calibration period to make a final estimate of stationary location (after Lagasse et al. 2022). The recalibration helped to account for individual and environmental variation in light levels, as it was derived from in-habitat data over a long time period, and produced location estimates that were more consistent with other information about wintering locations, such as resight and eBird data.

Post-processing

Locations produced by light-level geolocation are known to have a large degree of error (Lisovski et al., 2012). We removed locations above the Arctic circle, as 24-hour daylight is known to result in inaccurate locations (Lisovski, 2018). As birds on migration are not thought to commonly make major reversals in direction, we deleted locations where the angle between the bird’s original route and the reversal was less than 60 degrees (e.g. a complete reversal resulting in a bird backtracking along the same route would have an angle of 0°). In addition, we merged points within 250 km, with the position of the merged site weighted by the duration of stay at the original points, as points within this range cannot be reliably designated as separate locations (Clark et al., 2010; McKinnon et al., 2013).

Designation of wintering areas

In some individuals, discerning between migration stopover and wintering sites was clear: birds made short stops along migration, then stayed at a single site throughout the wintering period. However, many other individuals made significant movements within winter, during what is normally considered to be a stationary period; this type of intra-seasonal migration has become increasingly documented with improvements to tracking technology (McKinnon & Love, 2018). In these individuals, it can be less clear whether a stationary period represents wintering or a migratory stopover. To be consistent, we used the following criteria to identify wintering locations: any locations used during December-January, any locations further south than the December-January locations, and any locations within a certain distance of the December-January locations (500 km for birds wintering in Asia, North or Central America, 750 km for birds wintering in South America). The final wintering location for each individual was the last location that fit these criteria. These classification parameters represent estimates of distances that birds could fly without the major physiological preparations required for migratory movements (Piersma, 1998), and are based on natural groupings of movement distances within the wintering and migratory seasons. These criteria were developed through visual analysis of movement graphs to find parameters that generally selected what we considered to be biologically equivalent periods of wintering across all species.

S3: Determination of snowmelt values

At each breeding study site, a point situated in approximately the middle of the study site designated as the centre of a grid of 3 x 3 MODIS pixels, covering a total area of 2.25 km^2^. Where nests were >~8 km apart, we analysed snowmelt at multiple locations per site, and assigned each nest the value of the closest snow cover analysis site. The MODIS data provided daily estimates of percent snow cover for each of the 9 cells per 2.25 km^2^ site. We discarded pixels with cloud cover, and values indicating the MODIS algorithm could not determine the percent snow (the latter occurred very infrequently). Because of the coastal shape, in some cases cells indicated that they were >50% ocean; these cells were also discarded. We situated the central location to minimize discarding cells; a maximum of 3/9 cells were discarded at any site.

We calculated the median percent snow cover per site per day between April 1 and August 7. These dates were chosen to provide sufficient data before and after snowmelt to model the changes in snowmelt. Arctic snowfalls during the breeding season are not uncommon; we removed sudden 1-day spikes in snow cover that occurred in the middle or end of the breeding season, as these would have an exaggerated effect on the model, but would not affect the timing of nest initiation. Loss of snow coverage generally occurred quickly, with large sudden drops in coverage, therefore we used segmented models (Muggeo, 2008). We started by fitting a linear model with Julian date as the predictor and percent snow as the response variable. After determining the number of break points using the function *selgmented*, we used the function *segmented* on the linear model (Muggeo, 2008). We provided prior estimates for the location of the break point when not doing so resulted in a model with estimations outside of the accepted values (e.g., with % snow >100 or <0.). We used the models to estimate the daily value of percent snow coverage, bounded by 0 and 100, for each Julian day per year-location, which was then used to estimate the date at which snow cover was 50% (Smith et al., 2010). In rare cases, snowmelt fell below 50%, rose above 50%, then decreased again. In this case, we used the date of 50% snowmelt closest to the dates of nest initiation at that site.


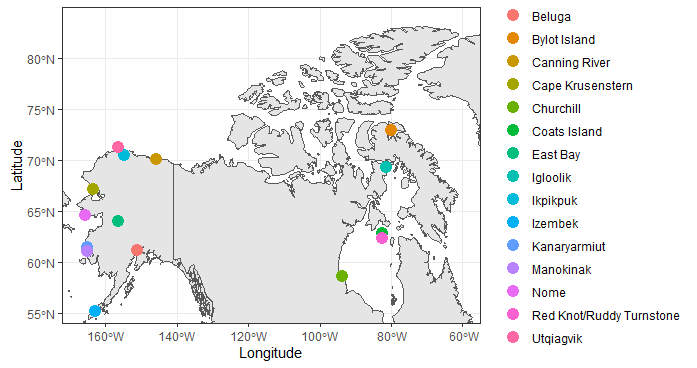


Figure 1. Breeding site locations. As the breeding sites of Red Knot and Ruddy Turnstone tagged away from the breeding grounds could not be estimated, one site in the eastern Canadian Arctic was used to judge relative timing in these species.

Table S1: Mean timing of migration and breeding by tag deployment site for six species of shorebirds. N = number of individuals tagged at the site; depart winter = mean Julian date of departure from the final wintering site; arrive breed = mean Julian date of estimated arrival to the breeding grounds; migration duration = mean number of days between departure from the final wintering site and arrival to the breeding site; nest initiation = mean Julian date of nest initiation date; pre-breeding = mean number of days between arrival to the breeding site and nest initiation; total migration distance = mean total great circle distance between the final wintering site and the breeding site, via stopover sites where birds remained for >48 hours, in kilometres. Standard deviations are given for each variable.

| Location | n | Depart winter ± SD | Arrive breed ± SD | Migration duration ± SD | Nest initiation ± SD | Pre-breeding ± SD | Total migration distance ± SD |
| --- | --- | --- | --- | --- | --- | --- | --- |
| Argentina | 2 | 66.0 (7.1) | 158.3 (n/a) | 87.3 (n/a) | 167.5 (2.1) | 7.7 (n/a) | 12,986.5 (975.2) |
| Beluga | 40 | 93.4 (7.3) | 121.9 (3.2) | 28.5 (7.6) | 131.6 (8.1) | 9.7 (8.4) | 13,946.8 (497.7) |
| Brazil | 25 | 136.6 (15.9) | 159.6 (3.5) | 22.8 (3.1) | 173.2 (7.9) | 12.1 (6.0) | 8679.0 (480.6) |
| Bylot Island | 20 | 112.1 (11.7) | 151.9 (4.2) | 93.9 (16.9) | 169.3 (4.6) | 17.3 (6.5) | 13,500.5 (445.3) |
| Cape Cod | 15 | 117.8 (34.6) | 160.4 (5.1) | 64.0 (38.0) | 173.3 (3.3) | 14.8 (9.2) | 3755.9 (881.4) |
| Cape Krusenstern | 14 | 113.1 (8.9) | 141.6 (4.9) | 28.5 (9.8) | 155.6 (5.3) | 14.0 (5.0) | 6440.0 (2209.5) |
| Canning River | 14 | 122.0 (13.7) | 149.5 (3.3) | 27.5 (15.4) | 161.9 (5.6) | 13.0 (5.7) | 8573.2 (1721.5) |
| Churchill | 27 | 112.1 (32.3) | 149.2 (5.2) | 37.1 (32.6) | 159.3 (7.5) | 10.3 (6.7) | 6551.8 (4315.0) |
| Coats Island | 10 | 123.1 (28.3) | 163.7 (5.4) | 40.6 (23.5) | 167.0 (1.7) | 7.4 (7.2) | 8740.7 (1627.1) |
| Delaware Bay | 9 | 124.0 (22.3) | 161.6 (5.4) | 43.0 (30.2) | 170.9 (4.4) | 9.7 (4.1) | 8589.8 (3185.6) |
| East Bay | 4 | 132.3 (2.1) | 159.4 (3.8) | 27.1 (5.8) | 174.0 (9.2) | 14.0 (5.1) | 7920.7 (929.6) |
| Florida | 7 | 131.0 (15.0) | 162.8 (4.1) | 25.5 (10.4) | 169.6 (2.4) | 6.2 (3.1) | 3015.3 (735.4) |
| Ikpikpuk | 6 | 101.3 (48.6) | 149.8 (4.1) | 48.5 (47.3) | 166.3 (6.7) | 16.5 (6.1) | 7822.3 (3392.9) |
| Izembek | 18 | 118 (5.4) | 129.3 (3.1) | 11.1 (5.4) | 141.1 (2.4) | 11.7 (3.2) | 4054.8 (346.9) |
| Kanaryarmiut | 4 | 115.5 (12.4) | 129.6 (8.1) | 14.1 (8.1) | 143.3 (12.7) | 13.6 (16.4) | 3153.4 (909.7 |
| Manokinak | 13 | 118.3 (17.4) | 134.1 (10.3) | 15.8 (19.6) | 148.8 (8.3) | 17.3 (7.6) | 3379.1 (524.0) |
| Nome | 2 | 68.5 (6.4) | 136.7 (n/a) | 72.7 (n/a) | 146.5 (2.1) | 8.3 (n/a) | 14,976.7 (281.2) |
| South Carolina | 7 | 111.6 (33.3) | 157.3 (1.1) | 22.8 (11.0) | 172.2 (5.3) | 16.9 (n/a) | 4736.6 (3907.2) |
| Texas | 31 | 138.5 (4.4) | 158.7 (6.3) | 20.0 (6.7) | 173.0 (3.9) | 13.2 (4.8) | 4276.9 (647.5) |
| Utqiaġvik | 43 | 114.9 (27.4) | 150.5 (3.9) | 35.6 (27.8) | 164.1 (6.5) | 13.6 (6.6) | 7365.1 (1890.4) |


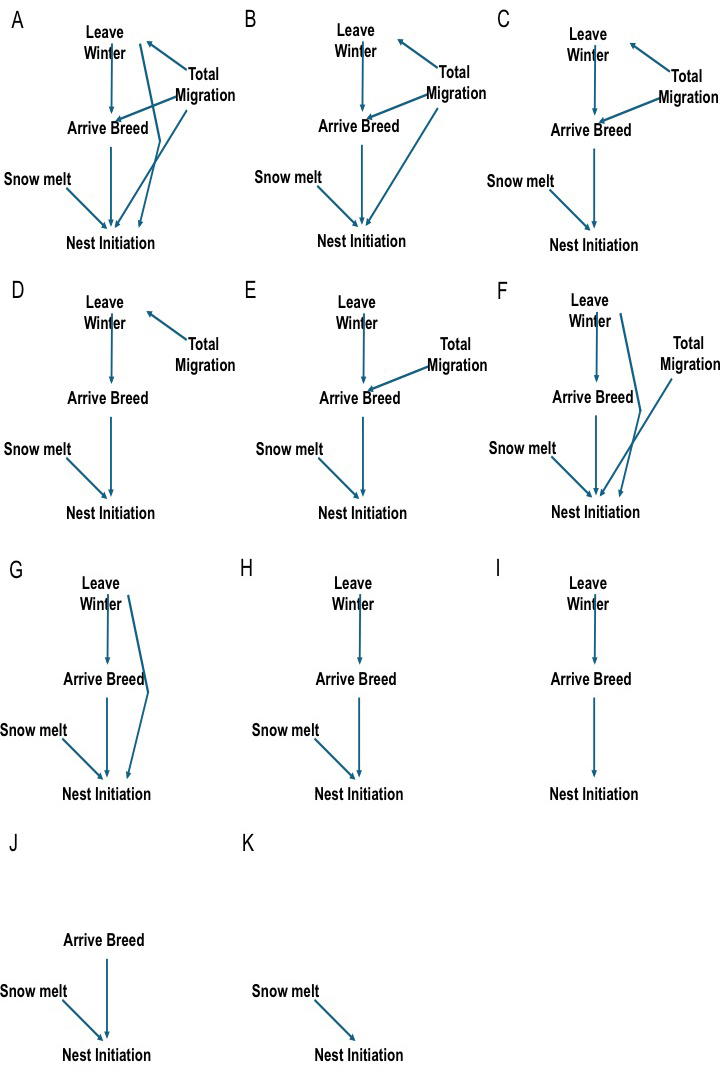


Figure 2. Candidate set of path analysis models. ‘Leave winter’ is the date an individual left the wintering grounds, ‘total migration’ is the distance in kilometres travelled by an individual between the final wintering site and the breeding site, via identified stopover sites, ‘Arrive breed’ is the estimated date when the bird arrived to the breeding grounds, ‘Snow melt’ is the date when 50% of ground is snow-free at the breeding site, and ‘Nest initiation’ is the date when the first egg was laid.

Table S2: Summary of the top (totalling to CICc weight 0.95) path models determining the relative influence of carry-over effects and local conditions on the timing of nest initiation. CICc weight for each model was calculated after models with uninformative variables were excluded. Standardized path coefficients and standard errors are provided for each variable; significant relationships are in bold. See Figure 1 for description of variables.

| Model Formula | CICc | Delta CICc | weight |
| --- | --- | --- | --- |
| All species (random = site + species) | | | |
| Leave winter ~ total migration  **-0.85±0.17**  Arrive breed ~ leave winter + total migration  0.07±0.04 **0.35±0.13**  Nest initiation ~ arrive breed + snow melt  **0.43±0.07 0.44±0.07** | 98.89 | 0.00 | 0.58 |
| Leave winter ~ tot total migration  **-0.85±0.16**  Arrive breed ~ leave winter  **0.15±0.06**  Nest initiation ~ arrive breed + snow melt  **0.50±0.06 0.42±0.06** | 99.50 | 0.61 | 0.42 |
| American Golden-Plover (random = site) | | | |
| Nest initiation ~ snow melt  **0.82±0.25** | 25.68 | 0.00 | 0.93 |
| arcticola Dunlin (random = site) | | | |
| Leave winter ~ total migration  **-0.65±0.11**  Arrive breed ~ leave winter  -0.11±0.15  Nest initiation ~ arrive breed + snow melt  0.29±0.15 0.25±0.15 | 49.31 | 0.00 | 1 |
| pacifica Dunlin (random = site) | | | |
| Arrive breed ~ leave winter + total migration  **0.31±0.08 0.70±0.11**  Nest initiation ~ arrive breed + snow melt  **0.33±0.14 0.45±0.14** | 33.52 | 0 | 1 |
| Hudsonian Godwit (random = site) | | | |
| Leave winter ~ total migration  **-0.40±0.13**  Arrive breed ~ leave winter + total migration  0.14±0.11 0.17±0.10  Nest initiation ~ arrive breed + snow melt  0.41±0.16 **0.41±0.13** | 37.98 | 0.00 | 0.76 |
| Leave winter ~ total migration  **-0.40±0.13**  Arrive breed ~ leave winter  0.06±0.10  Nest initiation ~ arrive breed + snow melt  **0.41±0.16 0.41±0.13** | 40.57 | 2.59 | 0.21 |
| Red Knot and Ruddy Turnstone (random = site and species) | | | |
| Leave winter ~ total migration  **-0.81±0.18**  Arrive breed ~ leave winter + total migration  0.17±0.18 0.29±0.21    Nest initiation ~ arrive breed  0.18±0.15 | 24.0974 | 0.00 | 0.53 |
| Leave winter ~ total migration  **-0.81±0.18**  Arrive breed ~ leave winter  0.03±0.15  Nest initiation ~ arrive breed  0.18±0.15 | 24.38 | 0.2817 | 0.46 |
